# Separation of Flexible Rod-like Particle Mixtures by Intersecting Air Flow

**DOI:** 10.3390/ma19050908

**Published:** 2026-02-27

**Authors:** Ashiq Ali, Gaoyan Shi, Yu Guo

**Affiliations:** 1 Department of Engineering Mechanics, Zhejiang University, Hangzhou 310027, China; ashiqali@zju.edu.cn; 2 State Key Laboratory of Clean Energy Utilization, Zhejiang University, Hangzhou 310027, China; 3 College of Design and Architecture, Zhejiang University of Technology, Hangzhou 310023, China; evashistudio@outlook.com

**Keywords:** separation by air flow, flexible particle, DEM-CFD, particle property, separator geometry

## Abstract

Air-induced separation of flexible rod-like particle mixtures in a specific separator is numerically investigated using a coupled Discrete Element Method (DEM) and Computational Fluid Dynamics (CFD) approach. In the separator, the mixture of flexible rod-like particles of different lengths and material densities deposits under the effect of gravity, and a horizontal airflow stream intersects the particle flow, blowing lighter particles in the mixture to translate horizontally and allowing the heavier ones to fall downwards. The model particles represent flexible biomass materials, specifically tobacco and stem particles. The initial packing density of the particle mixture is 8% by volume. The physical mechanism that causes particle segregation is analyzed. Subsequently, parametric studies are performed to examine the effects of some critical parameters on the extent of segregation, including inflow air velocities, initial particle packing density, volume fraction of heavier particles, particle size distribution, and flow field geometry. Finally, a suggestion is proposed to promote particle segregation in such a type of separator.

## 1. Introduction

Segregation and separation of granular materials with distinct physical properties of particles are crucial in many industries, including food processing, agriculture, energy, pharmaceuticals, and waste management. Extensive studies have been performed on the granular segregation and separation due to differences in particle shape [1,2], density [3,4], size [5,6], inelasticity [7,8], and surface roughness and friction [9,10]. In engineering practices, the differences in particle density and size are regarded as the most common mechanisms for particle segregation.

Particle segregation is undesirable in mixing processes of multiple particle components to achieve a well-mixed state. On the other hand, segregation can be utilized and promoted in purification, in which one particle component is separated from the rest in the mixture. For the purpose of promoting particle segregation and separation, which is the focus of the present work, various particle separator devices have been developed in many industrial processes, including wind-sifting separators, winnowing separators, and ballistic air separators [11,12,13].

In wind-sifting separating processes, the particles of different densities are usually separated using sieves. Additionally, zigzag wind-sifters are employed to separate the particles through air flows. These processes are widely employed in a variety of sectors, including recycling, metallurgy, chemicals, pharmaceuticals, furniture, food, and municipal solid waste, as well as coal processing [11,14]. Hagemeier et al. [15] numerically studied the separation effectiveness of a plot-scale zigzag wind-sifter separator using a coupled approach of Discrete Element Method (DEM) and Computational Fluid Dynamics (CFD). The effects of particle size, density, and air velocity were examined, and it was found that the separation performance varied as the process changed. The lighter/smaller particles have longer residence time than the heavier particles due to their susceptibility to slight variations in air velocity and the vortices. Roloff et al. [14] employed a multi-camera shadow-imaging system in a zigzag air separator to capture particle dynamics, and the experimental observation showed that air velocity had a greater impact on separation efficiency than particle loading rate.

Winnowing separators are widely used in agricultural processing and have two major types: air-screen separators and air-separators. In an air-screen separator, a combination of air current and vibrating screen is utilized to separate the granular mixtures. Zhao et al. [16] investigated whether incorporating a vibrating screen into separators decreased the mass of unwanted materials in rice; however, it would increase the loss rate of the rice due to entrainment. The DEM-CFD method has been used to numerically analyze the separation processes in the separators in the previous studies [17,18,19,20,21]. Yuan et al. [17] simulated the separation of rice-threshed mixtures flowing through a cylindrical sieve. The numerical results showed that the highest screening efficiency was obtained at an optimal air velocity, and in the present separator, air current played a dominant role in separating the materials of different physical properties. Ma et al. [18] simulated the rice-straw separation process under various airflow conditions (air velocities and flow directions). They found that an optimal air velocity of about 9 m/s led to the best rice purification, and a cyclone separator emerged as a prominent device for the rice-straw separation by maximizing the segregation due to the air current. He et al. [21] simulated the dynamics of soybeans and straws in the separator and thoroughly assessed the impacts of inlet/outlet air velocities on the purification performance of a bucket-free cyclone separator. Similarly, Dai et al. [19] analyzed cyclone separator performance for the purification of flax threshing materials based on the air-induced particle segregation. Ali et al. [20] simulated tobacco-stem particle separation processes within a winnower, in which the particle mixture horizontally flows in, and the air flows vertically to intersect with the particle flow. They found that the air velocity, material feed rate, and geometry of the equipment all affected the separating outcomes.

Recent studies highlight the importance of particle flexibility in granular flows. Wang et al. [22] reviewed the mechanical behavior of flexible fiber assemblies, noting that conventional theories for regular granular materials (e.g., dry sands) are unsuitable for fibers due to their elongation-induced energy dissipation. Zhou et al. [23] presented advanced CFD-DEM theory for multiphase flows with biogenic particles, simulating complex behaviors of varying biomass sizes and shapes. However, these flexibility models remain unintegrated into DEM-CFD separator simulations for biomass purification, leaving a critical gap in predicting separation performance for deformable materials.

In this work, we address this gap by using a coupled DEM-CFD method to simulate the separation of flexible rod-like particle mixtures in a unique process, where the particle mixture falls down under gravity into the separator through a vertical tube. In the process, the particle components of lower densities (desirable products) move horizontally, conveyed by a horizontal high-speed air stream, while the components of higher densities (impurities) keep falling down vertically into a collector. Such a particle separation process is usually encountered when purifying biomass materials, including tobacco particles and agricultural grains. Unlike previous studies that typically treat particles as rigid bodies, this study explicitly accounts for particle flexibility. Flexible particles deform during collisions, dissipating kinetic energy and reducing rebound velocities compared to rigid particles. This mechanical behavior is particularly relevant for biomass materials, which exhibit significant deformation subject to external loads. The deformation of a flexible particle affects the drag force on the particle. Thus, the particle motion and separation are altered by the particle flexibility. Incorporating particle flexibility provides more realistic segregation predictions and improves separator design reliability.

Thus, purification of the tobacco particles by removing stem particles is simulated as a representative case in the present study. The particle separation in a specific type of air separator, in which the downflow particle mixture is separated by a horizontal intersecting air current, is numerically investigated. Based on the numerical simulations, the effects of inflow air velocity, material feed rate, properties of the particle mixture, and separator geometry on the separation are explored, and some advice is provided to maximize separation performance for such separators.

## 2. The DEM-CFD Method

In the current simulations, a coupled DEM-CFD method is used, where flexible rod-like particles are modeled using a discrete element method (DEM)-based flexible fiber model, and the gas phase is resolved via a computational fluid dynamics (CFD) approach on fixed computational grids. The interaction between particles and air is treated using a two-way coupling scheme. The present in-house DEM-CFD code has been developed through the previous numerical studies on fluidization [24,25], pneumatic conveying [26], and air-induced segregation [20,27]. The theoretical aspects and governing equations of this numerical method are described below.

### 2.1. Flexible Fiber Model

**Figure 1:**
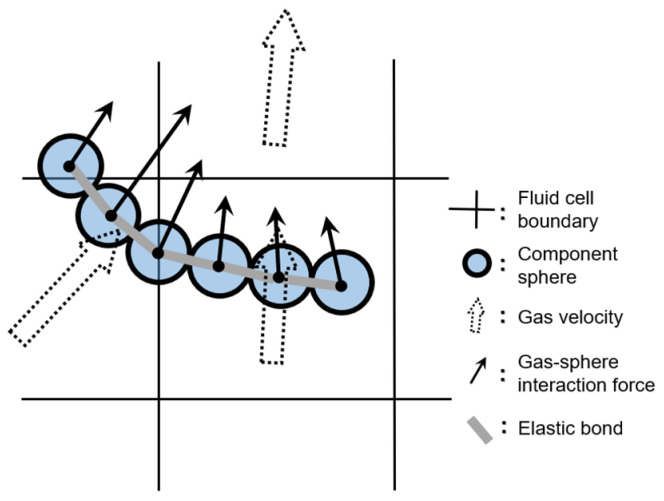
Schematic of hydrodynamic forces acting on a flexible fiber of aspect ratio (*AR*) 6; these forces are computed for each component sphere using local mean airflow variables.

In the simulations, a fiber is represented as a chain of identical spheres connected by elastic bonds (Figure 1). Inter-particle bonding forces and moments preserve connectivity between adjacent spheres while resisting deformation under external loads, expressed as [28]

(1) $$dF_{n}^{b}=K_{n}^{b}d\delta_{n}^{b}=\frac{E_{b}A}{l_{b}}d\delta_{n}^{b}=\frac{E_{b}A}{l_{b}}v_{n}^{r}dt,$$

(2) $$dF_{t}^{b}=K_{t}^{b}d\delta_{t}^{b}=\frac{G_{b}A}{l_{b}}d\delta_{t}^{b}=\frac{G_{b}A}{l_{b}}v_{t}^{r}dt,$$

(3) $$dM_{n}^{b}=K_{tor}^{b}d\theta_{n}^{b}=\frac{G_{b}I_{p}}{l_{b}}d\theta_{n}^{b}=\frac{G_{b}I_{p}}{l_{b}}\omega_{n}^{r}dt,$$

(4) $$dM_{t}^{b}=K_{ben}^{b}d\theta_{t}^{b}=\frac{E_{b}I}{l_{b}}d\theta_{t}^{b}=\frac{E_{b}I}{l_{b}}\omega_{t}^{r}dt,$$

where the incremental forces and moments in the bond are defined as follows: the normal force $dF_{n}^{b}$ and tangential force $dF_{t}^{b}$ correspond to incremental deformations $d\delta_{n}^{b}$ and $d\delta_{t}^{b}$ scaled by their respective stiffnesses $K_{n}^{b}$ and $K_{t}^{b}$. Similarly, the torsional moment $dM_{n}^{b}$ and bending moment $dM_{t}^{b}$ relate to incremental angles $d\theta_{n}^{b}$ and $d\theta_{t}^{b}$ via the torsional stiffness $K_{tor}^{b}$ and bending stiffness $K_{ben}^{b}$. The bond is modeled as a cylindrical element of radius $r_{b}$ and length $l_{b},$ with the cross-sectional area $A=\pi r_{b}^{2}$, polar area moment of inertia $I_{p}=\frac{\pi r_{b}^{4}}{2}$, and area moment of inertia $I=\frac{\pi r_{b}^{4}}{4}$. The bond radius is taken as equal to the radius of the constituent sphere ($r_{b}=r_{s})$ in the present model. The elastic properties of the bond are characterized by Young’s modulus $E_{b}$ and a shear modulus $G_{b}$, which are related via Poisson’s ratio ζ as

(5) $$E_{b}=2\left(1+\zeta \right)G_{b}.$$

The incremental bond deformations are computed by multiplying the time step dt by the relative velocities (both translational $v_{n}^{r}$, $v_{t}^{r}$ and rotational $\omega_{n}^{r}$, $\omega_{t}^{r}$) of two adjacent spheres.

The overall dynamics and deformation of a fiber emerge from the combined motion of its constituent spheres. The translational and rotational motions of each individual sphere are governed by Newton’s second law:

(6) $$m_{s}\frac{dv_{s}}{dt}=F^{c}+F^{b}+m_{s}g+F_{d}^{c}+F_{d}^{b}+F^{gs},$$

(7) $$J_{s}\frac{d\omega_{s}}{dt}=M^{c}+M^{b}+M_{d}^{c}+M_{d}^{b},$$

in which $m_{s}$ and $J_{s}=\frac{2}{5}m_{s}r_{s}^{2}$ represent the sphere’s mass and moment of inertia, respectively, while $v_{s}$ and $\omega_{s}$ are the translational and angular velocities. The sphere’s translational motion is governed by contact forces $F^{c}$ from non-bonded neighbors, bond forces $F^{b}$ (with normal $F_{n}^{b}$ and tangential $F_{t}^{b}$ components), and gravity $m_{s}g$. The moment $M^{c}$ results from the tangential component of contact forces. The bond moment $M^{b}$ includes contributions from the bond shear force $F_{t}^{b}$, twisting moment $M_{n}^{b}$, and bending moment $M_{t}^{b}$. The contact force $F^{c}$ is resolved into normal $F_{n}^{c}$ and tangential $F_{t}^{c}$ components. The normal force follows the Hertz model based on the current normal overlap between spheres [29]. The tangential force is governed by the Mindlin–Deresiewicz theory [30], which depends on the loading path, tangential displacement, and normal force, and is capped at the static friction limit $\mu F_{n}^{c}$ (where μ represents the friction coefficient). Complete implementation details are provided in [31].

Energy dissipation during collisions is incorporated through a contact damping force, $F_{d}^{c}$, introduced in Equation (6). Its normal and tangential components are expressed as [24]

(8) $$F_{dn}^{c}=-2\sqrt{\frac{5}{6}}\beta_{c}\sqrt{m_{f}^{*}K_{n}^{c}}\;v_{n}^{c},$$

(9) $$F_{dt}^{c}=-2\sqrt{\frac{5}{6}}\beta_{c}\sqrt{m_{f}^{*}K_{t}^{c}}\;v_{t}^{c},$$

where $m_{f}^{*}=\frac{m_{f1}m_{f2}}{m_{f1}+m_{f2}}$ is the effective mass of two contacting fibers with masses $m_{f1}$ and $m_{f2}$. The normal and tangential contact stiffnesses are defined as $K_{n}^{c}=\frac{dF_{n}^{c}}{d\delta_{n}}$ and $K_{t}^{c}=\frac{dF_{t}^{c}}{d\delta_{t}}$, respectively, with $\delta_{n}$ and $\delta_{t}$ denoting the normal and tangential overlap displacements at contact. The relative velocity components at the contact point are $v_{n}^{c}$ and $v_{t}^{c}$. The negative signs indicate that the damping force opposes relative motion. The parameter $\beta_{c}$ is the contact damping coefficient, which controls the rate of energy loss during collisions. The contact damping moment $M_{d}^{c}$ in Equation (7) arises from the tangential contact damping force $F_{dt}^{c}$.

To capture energy dissipation from elastic wave propagation during rapid fiber deformation, bond damping is introduced. The bond damping forces $F_{d}^{b}$ and bond moments $M_{d}^{b}$ exerted on component spheres are linearly proportional to the fiber deformation rates $v_{n}^{r}$, $v_{t}^{r}$, $\omega_{n}^{r}$, and $\omega_{t}^{r}$ [24]:

(10) $$F_{dn}^{b}=-\beta_{b}\sqrt{2m_{s}K_{n}^{b}v_{n}^{r}},$$

(11) $$F_{dt}^{b}=-\beta_{b}\sqrt{2m_{s}K_{t}^{b}v_{t}^{r}},$$

(12) $$M_{dn}^{b}=-\beta_{b}\sqrt{2J_{s}K_{tor}^{b}\omega_{n}^{r}},$$

(13) $$M_{dt}^{b}=-\beta_{b}\sqrt{2J_{s}K_{ben}^{b}\omega_{t}^{r}},$$

where $\beta_{b}$ is the bond damping coefficient.

Finally, there is the gas–solid interaction force $F^{gs}$ (Equation (6)) between gas and the component sphere, comprising pressure gradient, viscous, and drag contributions [32]:

(14) $$F^{gs}=-V_{s}\nabla p+V_{s}\nabla \cdot \tau_{g}+\varepsilon F_{drag},$$

where $V_{s}$ is the sphere volume, p is the local gas pressure, $\tau_{g}$ is the local viscous stress tensor for a Newtonian fluid, *ε* is the local porosity, and $F_{drag}$ is the drag force acting on the component sphere.

### 2.2. Gas-Phase Governing Equations

The conservation of mass and momentum for the gas flow is governed by the following equations [33]:

(15) $$\frac{\partial \left(\varepsilon \rho_{g}\right)}{\partial t}+\nabla \cdot \left(\varepsilon \rho_{g}u_{g}\right)=0,$$

(16) $$\frac{\partial \left(\varepsilon \rho_{g}u_{g}\right)}{\partial t}+\nabla \cdot \left(\varepsilon \rho_{g}u_{g}u_{g}\right)=-\nabla p+\nabla \cdot \tau_{g}-F_{V}+\varepsilon \rho_{g}g,$$

where $\rho_{g}$ is the gas density and $u_{g}$ is the gas velocity. The volumetric gas–fiber interaction force, $F_{V}$, is computed by summing the gas–sphere interaction forces $F^{gs}$ on all $n_{s}$ spheres within a given fluid cell and dividing by the fluid cell volume $V_{cell}$,

(17) $$F_{V}=\sum_{i=1}^{n_{s}}\frac{{\left(F^{gs}\right)}_{i}}{V_{cell}}.$$

where the index i denotes the *i*-th constituent sphere located in the fluid cell. The drag force $F_{drag}$ on a single sphere of $d_{s}$ follows the correlation by Di Felice [34]:

(18) $$F_{\mathrm{drag}\;}=\frac{1}{2}C_{D}\rho_{g}\frac{\pi d_{s}^{2}}{4}\varepsilon^{2}\left|u_{g}-v_{s}\right|\left(u_{g}-v_{s}\right)\varepsilon^{-\left(\chi +1\right)},$$

where $u_{g}-v_{s}$ is the relative velocity between the gas and sphere. The drag coefficient $C_{D}$ is defined as

(19) $$C_{D}={\left(0.63+\frac{4.8}{Re^{0.5}}\right)}^{2},$$

and the Reynolds number for the sphere, Re, is defined as

(20) Re=ρgdsε|ug−vs|μg,

with $\mu_{g}$ representing gas shear viscosity. The influence of neighboring solid particles is incorporated through the porosity function $\varepsilon^{-(\chi +1)}$, where the exponent χ varies with Reynolds number according to [34]:

(21) $$\chi =3.7-0.65\cdot \exp\left[-\frac{{\left(1.5-\log_{10}Re\right)}^{2}}{2}\right].$$

The Navier–Stokes Equations (15) and (16) are discretized using a semi-implicit finite difference scheme on a three-dimensional staggered Cartesian grid. Pressure and porosity are defined at cell centers, while velocity components are located on the faces of each fluid cell. Cell-centered fluid velocities are obtained by interpolating the face values.

### 2.3. Time Step

In the coupled DEM-CFD simulations, the critical time step necessary to maintain DEM stability is much smaller than that employed in the CFD framework, as described by Kafui et al. [32]. Therefore, the DEM-determined critical time step should be used for the coupled systems. Guo et al. [35] found that for flexible fiber DEM simulations, the time step size must be smaller than the time required for axial compressional or extensional waves to propagate across a single bond length $l_{b}$. The critical time step $\Delta t_{cri}$ is determined as

(22) $$\Delta t_{cri}=0.8165\cdot l_{b}\sqrt{\frac{\rho_{f}}{E_{b}}},$$

in which $\rho_{f}$ is the density of the fiber material. To ensure the numerical stability and accuracy, the real time step used in the simulations is conservatively chosen as a small fraction (less than 0.5) of $\Delta t_{cri}.$ The same time step is used for both the DEM and CFD, and the air–particle interaction forces are exchanged in each time step.

## 3. Numerical Model of Particle Separation in an Air Separator

**Figure 2:**
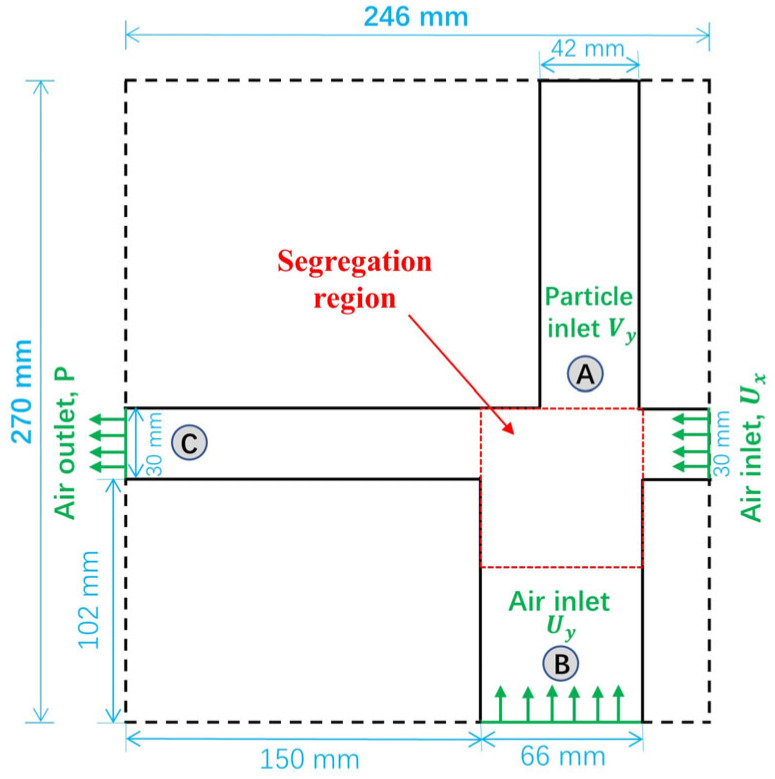
Computational domain for particle separation in an air separator, including particle inlet region (A), stem collector region (B), tobacco collector region (C), and a segregation region.

The geometry of the air separator is sketched in Figure 2. A rectangular domain of length × height × depth = $246\times 270\times 24\;mm^{3}$ is partitioned into several regions: the particle inlet region (A), the stem collector region (B), the tobacco collector region (C), and a segregation region at the cross of the above three regions. A mixture of stem and tobacco particles, which are the model particles in the present simulations, is randomly generated with an almost uniform distribution in Region A. In the separation process, the particle mixture falls under gravity. Meanwhile, a strong air current at a specified velocity of $U_{x}$ flows horizontally into the domain from the right-hand side boundary. The horizontal air flow interacts with the vertical particle flow in the separation region, resulting in most tobacco particles (with a lower material density of 264 kg/m^3^) traveling horizontally to the tobacco collector region (Region C) and most stem particles (with a higher density of 639.8 kg/m^3^) settling into the stem collector region (Region B) at the lower position. From the bottom boundary, air flows upward at a velocity of $U_{y}$ to prevent the tobacco particles from falling into Region B, creating a better chance for the tobacco particles to move horizontally to Region C. In Figure 2, solid black lines denote impermeable boundaries: no-slip walls applied to the gaseous phase and frictional walls interacting with the particulate phase. To reduce computational cost, a thin-sliced model of a depth of 24 mm is used in the present simulations, and periodic boundary conditions for both air and particle dynamics are assigned in the depth direction (perpendicular to the page in Figure 2). This simplification of the numerical model is reasonable considering the more dominant motions of the air and particles in the length and height directions than in the depth direction. The properties and boundary conditions of the airflow are listed in Table 1.

The Reynolds numbers are $Re_{h}\approx 7134$ at the horizontal air inlet (30 × 24 mm^2^ cross-section) with an air velocity of $U_{x}=4.0\;m/s$ and $Re_{v}\approx 8711$ at the vertical air inlet (66 × 24 mm^2^ cross-section) with an air velocity of $U_{y}=3.7\;m/s$. These values indicate turbulent airflow at both inlets. Therefore, the *k*-*ε* model is used to close the Reynolds-Averaged Navier–Stokes (RANS) equations to consider the effect of the turbulence.

**Figure 3:**
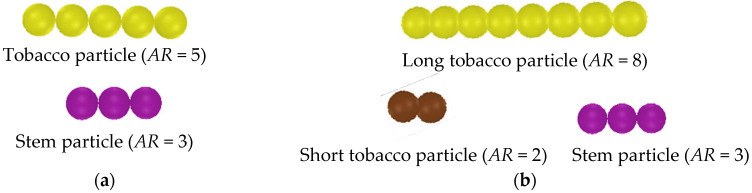
Bonded-sphere models of tobacco and stem particles used in the simulations: (**a**) particles in binary mixtures and (**b**) particles in ternary mixtures.

Present simulations consider binary and ternary mixtures of tobacco and stem particles. In the binary case (Figure 3a), tobacco particles are modeled using five bonded spherical elements of 2.7 mm diameter, corresponding to an aspect ratio of 5, whereas a stem particle consists of three bonded spheres, resulting in an aspect ratio of 3. The ternary mixture (Figure 3b) contains long tobacco particles with *AR* = 8 and short tobacco particles with *AR* = 2, and stem particles with *AR* = 3. The constituent spheres in these particles have the same diameter of 2.7 mm. Material densities were measured experimentally to be 639.8 kg/m^3^ for the stem and 264 kg/m^3^ for tobacco particles [27,36]. The elastic modulus $E_{b}$ associated with the bending of a tobacco particle was calibrated by fitting simulated packing densities to experimental data [36]. Since the impact of Poisson’s ratio *ζ* on particle contact interactions and deformation is very limited, a value of *ζ* = 0.2 is adopted, which is consistent with many biomass materials [27]. The shear modulus $G_{b}$ associated with the bending of the tobacco particle is obtained using

(23) $$G_{b}=\frac{E_{b}}{2\left(1+\zeta \right)}.$$

Owing to their material properties, tobacco particles exhibit substantially greater flexibility than stem particles, with differences of approximately five orders of magnitude in shear and elastic moduli governing bond twisting, shearing, and bending. The relevant properties of the particles are summarized in detail in Table 2. In the simulations, both the stem particle volume concentration in the mixture and the initial packing density of the mixture within the particle inlet region (Region A) are varied to examine their effects on separation performance.

In the present air separation process, air–particle interactions are more dominant than particle–particle interactions. Thus, the damping coefficients (in the range of 1.0 × 10^−3^–1.0 × 10^−1^), which affect the particle–particle contact forces, have limited impacts on the separation results. In the gas-fluidized bed simulations with the present flexible particle model [24], the thickness of the periodic thin slice is varied between $8d_{s}$ and 17$d_{s}$ ($d_{s}$ is the diameter of the component sphere in a composite particle), and the particle flow patterns and air pressure drops are insensitive to the slice thickness of the domain. Thus, in the present work, the thickness of the thin-slice domain is specified as 24 mm, i.e., $8.9d_{s}$.

The DEM modeling of mechanical responses of a single flexible particle is verified in [35] for tensile/compressive, bending, and twisting deformations. To validate the drag model and numerical DEM-CFD scheme for the simulations of gas-flexible-particle two-phase flows, the air pressure drops and air-induced segregation in gas-fluidized beds of the flexible fibers and spheres have been analyzed [24,27]. The simulation results are in good agreement with the previous experimental and numerical results.

## 4. Results and Discussion

Based on the simulation results, the mechanism that governs separation in the air separator is discussed. Thereafter, systematic studies are performed to examine the effects of key operating parameters (inflow air velocities and initial packing density), tobacco particle size distribution, components of the particle mixture, and geometrical structures of the separator.

### 4.1. Separation Mechanism by Air Flow

**Figure 4:**
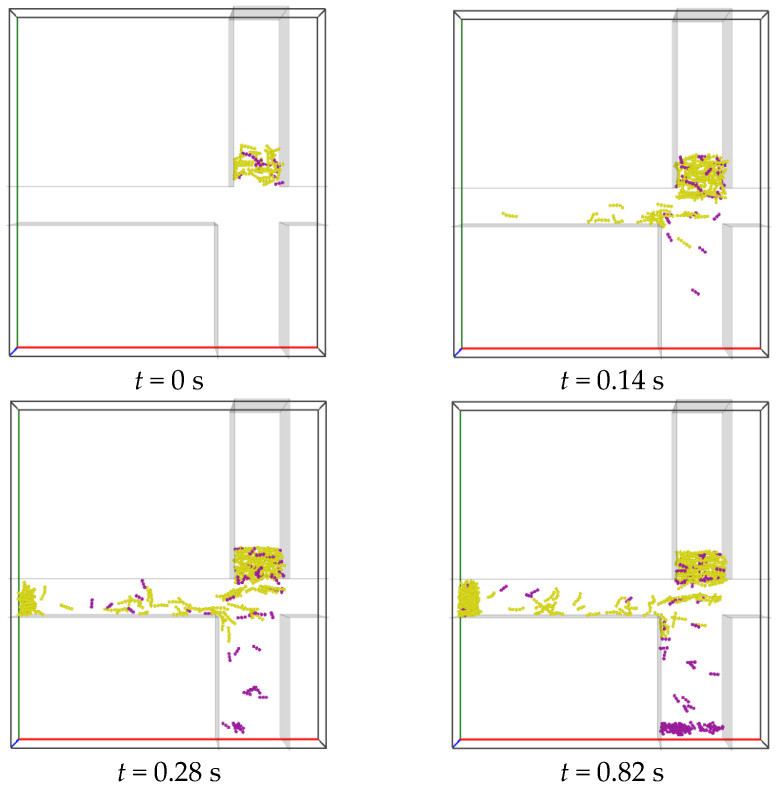
Snapshots of stem (magenta) and tobacco (yellow) particles in the separator at different times. The horizontal and vertical air velocities are specified as $U_{x}=4.0\;m/s$ and $U_{y}=3.7\;m/s$, respectively. The mixture has an initial packing density of 8% and a stem particle volume fraction of 17%.

Snapshots obtained from the simulations of tobacco-stem particle separation are shown in Figure 4. The volume concentration of stem particles in the mixture is specified as 17%. The mixture of particles is generated at a specified rate in Region A, allowing continuous particle flows from Region A to the Segregation region (Figure 2). The air inflow velocities are set to $U_{x}=4.0\;m/s$ (right-hand inlet) and $U_{y}=3.7\;m/s$ (bottom boundary of Region B). The particles with zero initial velocities fall under the effect of gravity into the Segregation region, where segregation occurs, induced by air. Eventually, tobacco particles (yellow) transport horizontally to Region C (tobacco collector), and the stem particles (magenta) settle in Region B (stem collector).

Assuming that a particle takes a duration of Δt to pass vertically through the Segregation region and the particle has an average horizontal velocity of $v_{h}$, the horizontal displacement of the particle within the Segregation region is $S_{h}=v_{h}\Delta t$. Thus, the criterion for the particle to reach the horizontal channel on the left-hand side (Figure 2) is that its horizontal displacement should satisfy $S_{h}>S_{0}$, in which $S_{0}$ is a critical displacement depending on the geometry of the Segregation region. Compared to the stem particles (with higher density), the tobacco particles (with lower density) have larger ratios of the vertical air drag force to the gravitational force, i.e., $F_{drag}^{y}/G$, resulting in a longer duration Δt for the tobacco particles residing in the Segregation region. In addition, the tobacco particles possess larger ratios of the horizontal air drag force to the particle mass (horizontal acceleration), i.e., $F_{drag}^{x}/m$, and thus higher horizontal velocity $v_{h}$. As a result, the tobacco particles travel by larger horizontal displacements $S_{h}$ than the stem particles in the separation process. Therefore, the tobacco particles gain a better chance to move into the horizontal channel on the left-hand side and are eventually collected in Region C. To achieve a good separation result, the horizontal displacements of the tobacco particles should satisfy $S_{h}>S_{0}$, while the stem particles satisfy $S_{h}<S_{0}$, allowing them to end up in the lower stem collector (Region B).

### 4.2. Effect of Horizontal Air Velocity

**Figure 5:**
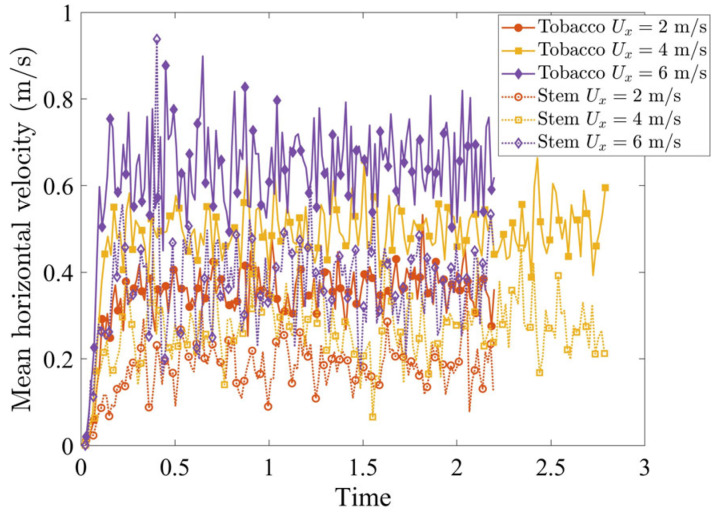
Mean horizontal velocity of tobacco and stem particles in the segregation region for various horizontal inflow air velocities $U_{x}$.

The horizontal air velocity $U_{x}$ governs the driving forces acting on the particles in the segregation region and controls particle trajectories toward either the stem collector (Region B) or the tobacco collector (Region C). Figure 5 shows the mean horizontal velocity of tobacco and stem particles in the segregation region varying with time. For both particle types, the particle velocity increases monotonically with $U_{x}$, while the tobacco particles consistently gain higher mean horizontal velocities than the stems at the same $U_{x}$. This velocity difference is the fundamental mechanism that induces the segregation.

**Figure 6:**
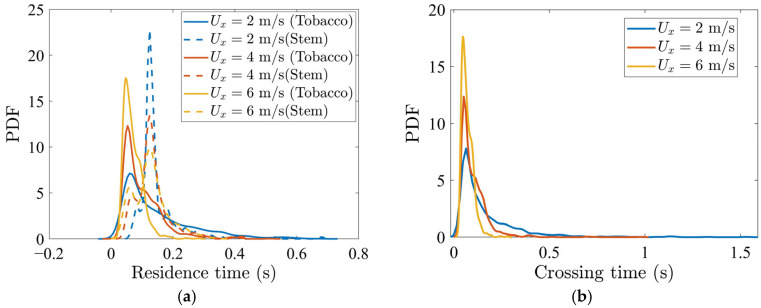
(**a**) PDF distribution of residence time of tobacco and stem particles in the segregation region and (**b**) PDF distribution of tobacco particle crossing time (the time duration it takes for a tobacco particle to cross the gap and reach Region C), for various horizontal inflow air velocities $U_{x}$.

The consequences of these velocity differences are reflected in the temporal statistics of particle transport. The probability density functions (PDFs) of residence time in the segregation region (Figure 6a) exhibit three systematic trends with increasing $U_{x}$: (i) the peak shifts to shorter times, (ii) the distribution narrows, and (iii) the disparity between tobacco and stem particles widens. At $U_{x}=2.0\;m/s$, the tobacco particles display broader residence-time distributions with long tails, indicating that a substantial fraction experiences prolonged, erratic transport. At $U_{x}=6.0\;m/s$, the tobacco particles show a narrower distribution and a higher peak, and thus nearly all tobacco particles pass the segregation region within a shorter time duration. The stem particles, while also responding to higher $U_{x}$, retain broader distributions and longer mean residence times than the tobacco particles, due to their greater inertia.

For the tobacco particles specifically, the PDF of crossing time (the time duration required to cross the gap and reach Region C) is plotted for various $U_{x}$ in Figure 6b. The peak occurs at a shorter duration and increases as $U_{x}$ increases. Thus, a higher horizontal air velocity reduces the crossing time for the tobacco particles.

**Figure 7:**
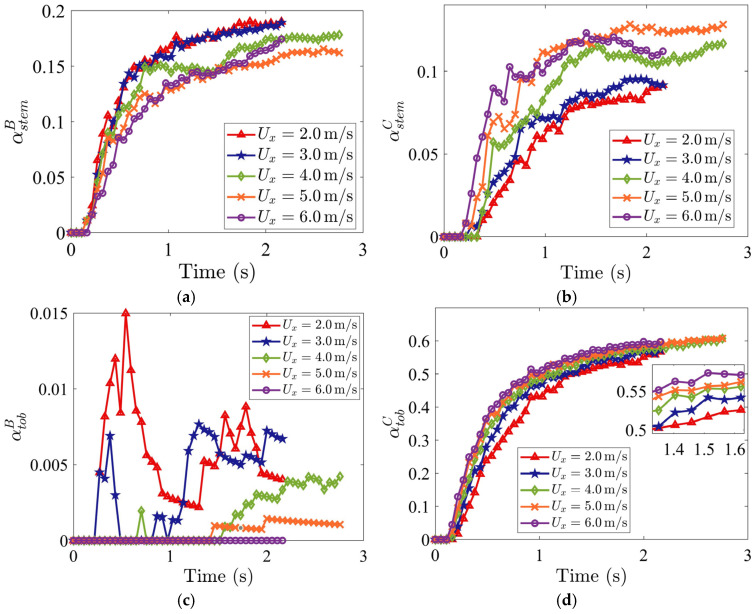
Time evolutions of mass fractions (**a**) $\alpha_{stem}^{B}$ (stem in stem collector), (**b**) $\alpha_{stem}^{C}$ (stem in tobacco collector), (**c**) $\alpha_{tob}^{B}$ (tobacco in stem collector), and (**d**) $\alpha_{tob}^{C}$ (tobacco in tobacco collector), for various horizontal inflow air velocities $U_{x}$. The vertical air velocity is specified as $U_{y}=3.7\;m/s$. The volume fraction of stem particles is 17%, and the initial packing density is 8%.

The integrated outcome of these velocity-driven mechanisms is quantified by the time evolution of tobacco and stem particle mass fractions in the stem collector (Region B) and tobacco collector (Region C), as shown in Figure 7. The stem particle mass fraction in Region B is defined as $\alpha_{stem}^{B}=m_{stem}^{B}/m_{total}^{domain}$ where $m_{stem}^{B}$ is the mass of stem particles in Region B and $m_{total}^{domain}$ is the total mass of all the particles in the entire domain. The stem particle mass fraction in Region C is defined as $\alpha_{stem}^{C}=m_{stem}^{C}/m_{total}^{domain}$ where $m_{stem}^{C}$ is the mass of stem particles in Region C. Similarly, the tobacco particle mass fraction in Regions B and C, represented by $\alpha_{tob}^{B}$ and $\alpha_{tob}^{C}$, are defined as $\alpha_{tob}^{B}=m_{tob}^{B}/m_{total}^{domain}$ and $\alpha_{tob}^{C}=m_{tob}^{C}/m_{total}^{domain}$, respectively, in which $m_{tob}^{B}$ is the mass of tobacco particles in Region B and $m_{tob}^{C}$ is the mass of tobacco particles in Region C. These mass fractions are dimensionless quantities that range from 0 to 1. A mass fraction of $\alpha_{stem}^{B}=1$ indicates the complete collection of stems in Region B, whereas $\alpha_{stem}^{B}=\;$0 indicates no collection of stems in Region B.

As $U_{x}$ increases, both stem and tobacco mass fractions in the stem collector ($\alpha_{stem}^{B}$ and $\alpha_{tob}^{B})$ decline (Figure 7a,c), because the larger drag forces on the particles by the horizontal air stream prevent them from falling down into the stem collector. At $U_{x}=\;$6.0 m/s, $\alpha_{tob}^{B}$ is nearly zero, meaning the loss of tobacco product in the stem collector is eliminated. Conversely, both the stem and tobacco particle mass fractions in Region C, $\alpha_{stem}^{C}$ and $\alpha_{tob}^{C}$, increase with increasing $U_{x}$, due to the effect of stronger air drag, as shown in Figure 7b,d.

**Figure 8:**
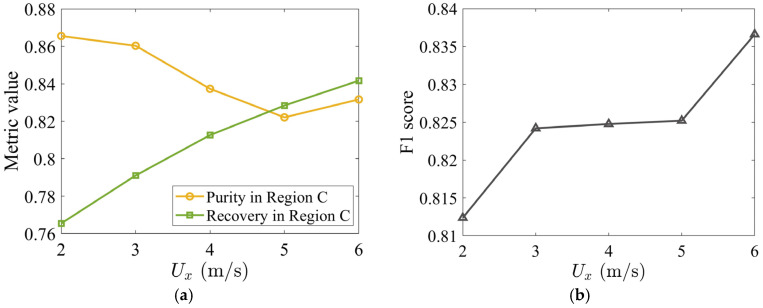
(**a**) Time-averaged tobacco purity and recovery in Region C as a function of $U_{x}$ and (**b**) corresponding F1 score. All metrics were computed by time-averaging over the steady-state interval t=[1.0, 2.2] s. The vertical air velocity is specified as $U_{y}=3.7\;m/s$. The volume fraction of stem particles is 17%, and the initial packing density is 8%.

To evaluate this trade-off quantitatively, time-averaged tobacco purity and recovery in Region C are computed over the steady-state interval t=[1.0,2.2]s. The purity, $P_{C}$, defined as $P_{C}=\langle m_{tob}^{C}\rangle /(\langle m_{tob}^{C}\rangle +\langle m_{stem}^{C}\rangle )$, measures tobacco concentration in Region C. The recovery, $R_{C}$, defined as $R_{C}=\langle m_{tob}^{C}\rangle /\langle m_{tob}^{domain}\rangle$, represents the fraction of total tobacco particles that have been successfully collected in Region C. As $U_{x}$ increases from 2 to 6 m/s, the purity declines slightly from 86.5% to 83.2% (Figure 8a). However, the recovery improves significantly from 76.5% to 84.2% over the same range, confirming that the suppression of tobacco loss to the stem collector outweighs the stem contamination.

The F1 score, a harmonic mean of purity and recovery ($F1=2P_{C}R_{C}/(P_{C}+R_{C})$), provides a single metric for identifying the optimal operating condition (Figure 8b). The F1 score increases monotonically with $U_{x}$ and reaches its maximum of 83.7% at $U_{x}=6\;m/s$. Hence, within the range examined, the highest horizontal velocity yields the best overall balance between product purity and recovery.

### 4.3. Effect of Vertical Air Velocity

The vertical air velocity $U_{y}$ (imposed at the bottom boundary of Region B, Figure 2) generates an upward drag force that counteracts gravity. By partially suspending particles in the segregation region, $U_{y},$ prolongs their exposure to the horizontal cross-flow, and thereby influences whether they settle into the stem collector (Region B) or are carried toward the tobacco collector (Region C).

**Figure 9:**
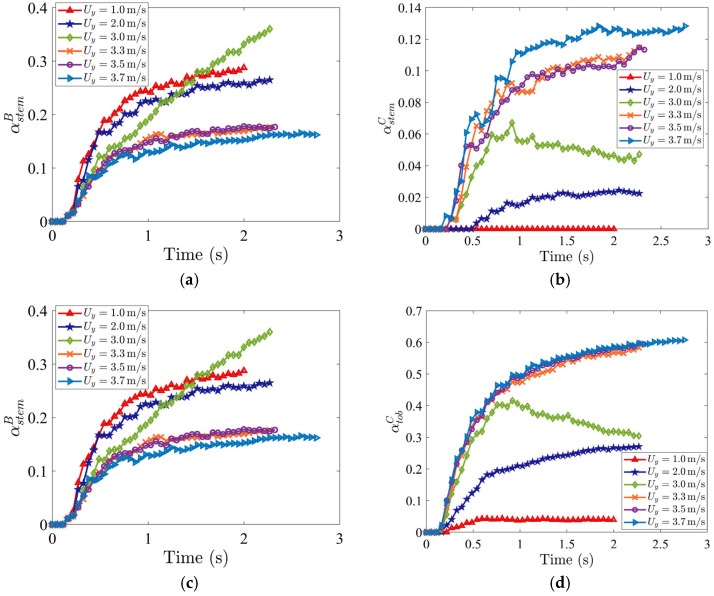
Time evolutions of mass fractions (**a**) $\alpha_{stem}^{B}$ (stem in stem collector), (**b**) $\alpha_{stem}^{C}$ (stem in tobacco collector), (**c**) $\alpha_{tob}^{B}$ (tobacco in stem collector), and (**d**) $\alpha_{tob}^{C}$ (tobacco in tobacco collector), for various vertical inflow air velocities, $U_{y}$. The horizontal air velocity is specified as $U_{x}=5.0\;m/s$. The volume fraction of stem particles is 17%, and the initial packing density is 8%.

Figure 9a,c show that the mass fractions of both stem and tobacco particles in Region B, $\alpha_{stem}^{B}$ and $\alpha_{tob}^{B}$, decrease by increasing the vertical air inlet velocity $U_{y}$. Higher upward drag prevents particles from falling into Region B, keeping them suspended where the horizontal flow can transport them further. Figure 9b,d indicate that the mass fractions of both stem and tobacco particles in Region C, $\alpha_{stem}^{C}$ and $\alpha_{tob}^{C}$, increase with increasing $U_{y}$. At the highest $U_{y}$ tested (3.7 m/s), maximum tobacco particles reach Region C, and so tobacco loss to the stem collector is nearly eliminated.

**Figure 10:**
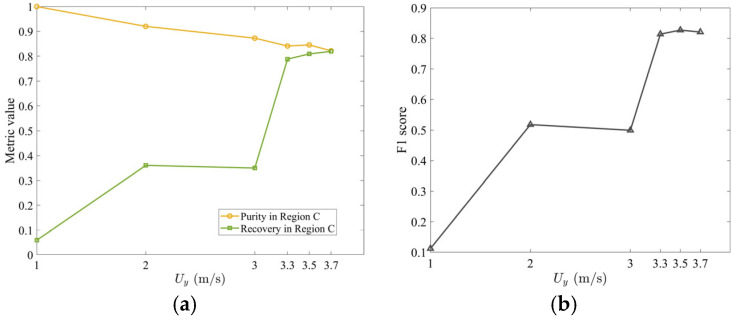
(**a**) Time-averaged tobacco purity and recovery in Region C as a function of $U_{y}$ and (**b**) corresponding F1 score. All metrics were computed by time-averaging over the steady-state interval t=[1.0, 2.0] s. The horizontal air velocity is specified as $U_{x}=5.0\;m/s$. The volume fraction of stem particles is 17%, and the initial packing density is 8%.

The improved recovery of tobacco, however, comes at the cost of increased stem carryover. This trade-off is quantified in Figure 10a, which shows that increasing vertical air velocity $U_{y}$ from 1 to 3.7 m/s causes tobacco purity in Region C to drop from 100% (no stem contamination at $U_{y}=1.0\;m/s)$ to 81.9% at $U_{y}=3.7\;m/s$. Meanwhile, recovery improves drastically from only 5.0% to 81.9%. The vertical velocity thus transforms the separator from a device that collects negligible tobacco (but with perfect purity) into one that captures most of the tobacco (though with modest stem contamination). To identify the optimal balance, the F1 score (Figure 10b) plateaus near its peak of ~82.6% across 3.3–3.7 m/s, with the optimum at 3.5 m/s.

### 4.4. Effect of Initial Packing Density

The initial packing density of the particle bed, Φ, refers to the volume fraction occupied by the particle bed in Region A. It affects interstitial airflow and particle contacts, and therefore, may impact segregation. This study examines three packing densities of Φ=4%,8%,and 16%.

To assess segregation, normalized mass fractions of tobacco and stem particles in Regions B and C are analyzed. The normalized mass fractions of tobacco particles in Regions B and C are defined as normalized $\alpha_{tob}^{B}={\alpha_{tob}^{B}/\alpha }_{tob}^{ave}$ and normalized $\alpha_{tob}^{C}={\alpha_{tob}^{C}/\alpha }_{tob}^{ave}$, respectively, where $\alpha_{tob}^{ave}=m_{tob}^{domain}/m_{total}^{domain}$, $m_{tob}^{domain}$ is the mass of tobacco particles in the entire domain, and $m_{total}^{domain}$ is the total mass of the mixture in the entire domain. Similarly, the normalized mass fractions of stem particles in Regions B and C are defined as $\alpha_{stem}^{B}=\alpha_{stem}^{B}/\alpha_{stem}^{ave}$ and $\alpha_{stem}^{C}=\alpha_{stem}^{C}/\alpha_{stem}^{ave}$, respectively, where $\alpha_{stem}^{ave}=m_{stem}^{domain}/m_{total}^{domain}$ and $m_{stem}^{domain}$ is the mass of stem particles in the entire domain. The normalized mass fractions provide an unbiased comparison and highlight the relative degree of segregation regardless of the absolute quantities at different packing densities.

**Figure 11:**
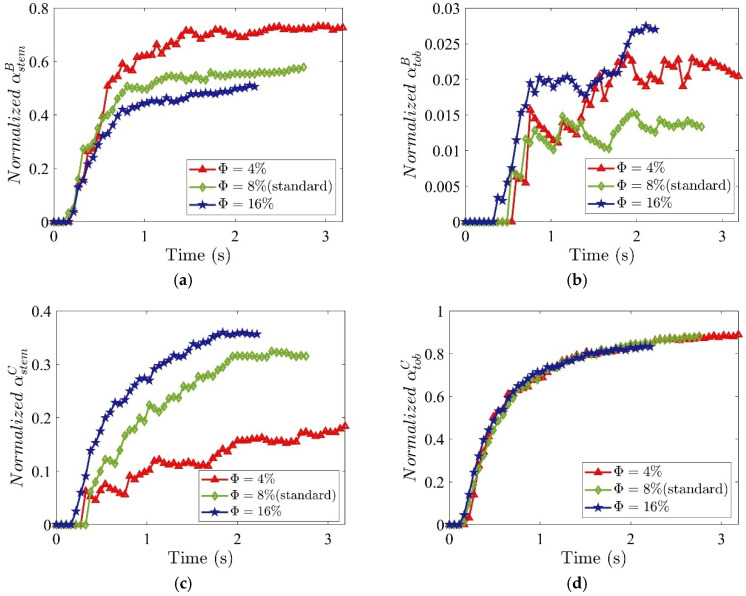
Time evolution of normalized mass fraction of (**a**) stem in Region B, (**b**) tobacco in Region B, (**c**) stem in Region C, and (**d**) tobacco in Region C, for different initial packing densities Φ. The horizontal and vertical air velocities are specified as $U_{x}=4.0\;m/s$ and $U_{y}=3.5\;m/s$, respectively. The volume fraction of stems in the mixture is 17%.

**Figure 12:**
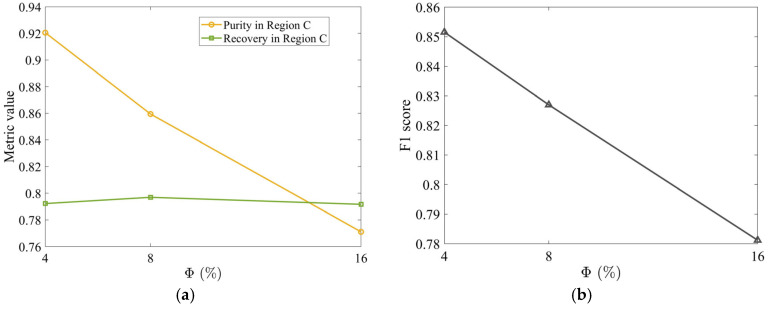
(**a**) Time-averaged tobacco purity and recovery in Region C as a function of Φ and (**b**) corresponding F1 score. All metrics were computed by time-averaging over the steady-state interval t=[1.0, 2.2] s. The horizontal and vertical air velocities are specified as $U_{x}=4.0\;m/s$ and $U_{y}=3.5\;m/s$, respectively. The volume fraction of stems in the mixture is 17%.

As Φ increases, the normalized stem mass fraction in Region B decreases (Figure 11a), but increases in Region C (Figure 11c), degrading tobacco purity. Meanwhile, the normalized tobacco mass fraction in Region B (Figure 11b) indicates a greater loss of tobacco, while it remains nearly unchanged in Region C (Figure 11d). These findings demonstrate that higher Φ deteriorates segregation performance, reducing purity without affecting recovery (Figure 12a). Denser packing restricts particle mobility, hindering separation. Consequently, a sparse initial bed maximizes performance by providing sufficient free volume for particles to respond individually to the aerodynamic field, minimizing both stem carryover and tobacco loss. The F1 score peak (Figure 12b) confirms a lower initial packing density of Φ = 4% as optimal, balancing 92.0% purity with 79.2% recovery.

### 4.5. Effect of Volume Fraction of Stem Particles in the Mixture

**Figure 13:**
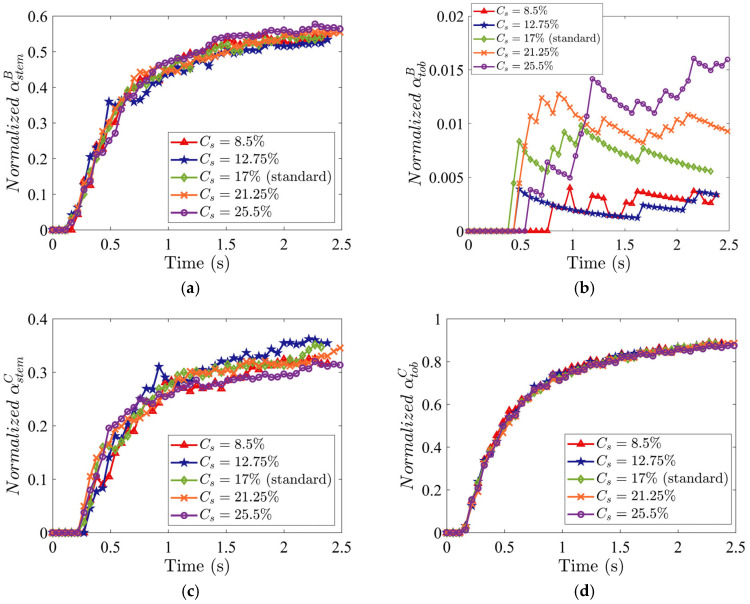
Time evolution of normalized mass fraction of (**a**) stem in Region B, (**b**) tobacco in Region B, (**c**) stem in Region C, and (**d**) tobacco in Region C, for different stem concentrations $C_{s}$. The horizontal and vertical air velocities are specified as $U_{x}=5.0\;m/s$ and $U_{y}=3.5\;m/s$, respectively. The initial packing density is 8%.

The volume concentration of stem particles in the mixture is defined as $C_{s}=V_{s}/V_{mix}$, where $V_{s}$ is the stem volume and $V_{mix}$ is the mixture volume. The normalized fractions $\alpha_{stem}^{B}$, $\alpha_{stem}^{C}$, and $\alpha_{tob}^{C}$ are weakly dependent on $C_{s}$, as shown in Figure 13a,c,d. However, $\alpha_{tob}^{B}$ increases with increasing $C_{s}$ (Figure 13b), indicating greater tobacco particle losses into the stem collector. At higher $C_{s}$, stems increasingly dominate the inter-particle interactions and hinder horizontal transport of lighter tobacco particles through collisions and by locally disturbing the airflow, causing some tobacco to be dragged downward.

**Figure 14:**
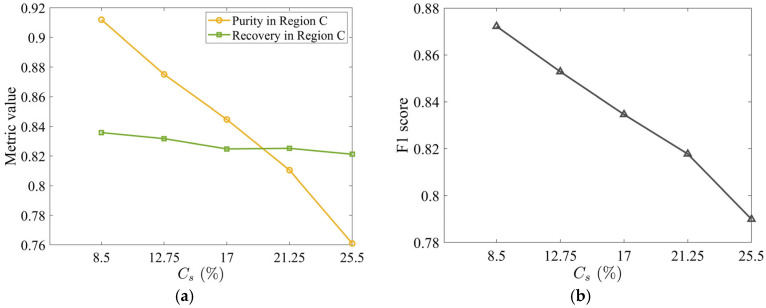
(**a**) Time-averaged tobacco purity and recovery in Region C as a function of $C_{s}$ and (**b**) corresponding F1 score. All metrics were computed by time-averaging over the steady-state interval t=[1.0, 2.4] s. The horizontal and vertical air velocities are specified as $U_{x}=4.0\;m/s$ and $U_{y}=3.5\;m/s$, respectively. The volume fraction of stems in the mixture is 17%.

The increased tobacco loss at higher $C_{s}$ directly erodes the tobacco purity: time-averaged tobacco purity in Region C falls from 91% to 76% as $C_{s}$ increases from 8.5% to 25.5%, while tobacco recovery remains stable (Figure 14a). Thus, higher stem concentration harms purity exclusively. The F1 score is maximum at the lowest stem concentration, $C_{s}=8.5\%$ (Figure 14b), confirming that the minimal initial stem content optimizes the purity-recovery balance.

### 4.6. Separation of Ternary Mixture Versus Binary Mixture

**Figure 15:**
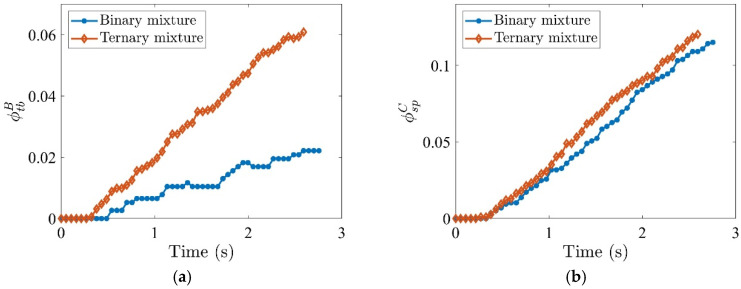
Time evolution of volume fractions (**a**) $\phi_{tb}^{B}$ (tobacco in stem collector) and (**b**) $\phi_{sp}^{C}$ (stem in tobacco collector) for binary and ternary mixtures. The inflow air velocities are specified as $U_{x}=4.0\;m/s$ and $U_{y}=3.5\;m/s$. The volume fraction of stem particles is 17%, and the initial packing density is 8%.

To explore the impact of tobacco size distribution on segregation, the results from the ternary mixture (Figure 3b) are compared with the binary mixture (Figure 3a). The binary mixture contains uniform tobacco particles (*AR* = 5), while the ternary mixture contains two sizes (*AR* = 8 and 2). The tobacco particle volume fraction in Region B, $\phi_{tb}^{B}$, increases over time for both mixtures, but at a higher rate of increase for the ternary mixture (Figure 15a), due to the shorter tobacco particles (*AR* = 2) experiencing less suspension by the vertical air current and falling more quickly. The stem particle volume fraction in Region C, $\phi_{sp}^{C}$, is also slightly higher for the ternary mixture (Figure 15b) as the stem particles are entrained by the falling tobacco particles. These higher fractions in both regions indicate poorer segregation performance for the ternary mixture.

### 4.7. Effect of Geometry of Separator

#### 4.7.1. Staggered Distance on Trailing Side $\epsilon_{R}$

**Figure 16:**
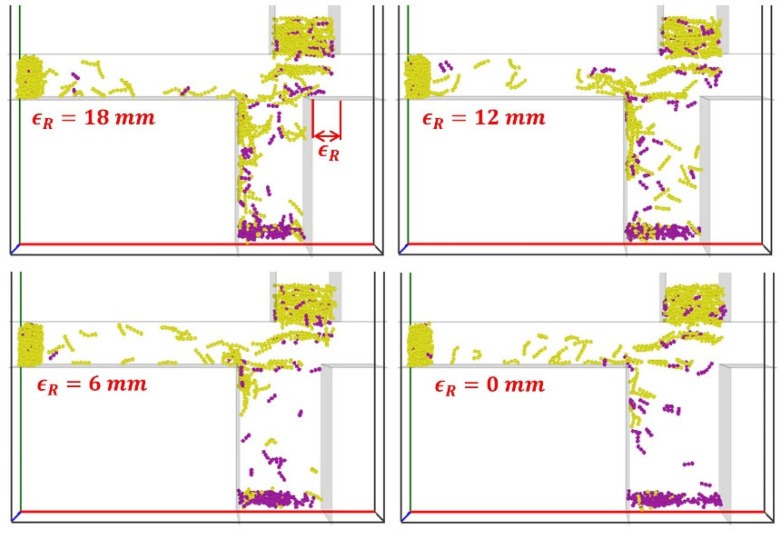
Snapshots of tobacco-stem particle segregation for distinct values of the staggered distance on the trailing side $\epsilon_{R}$ with $U_{x}=5.0\;m/s\;and\;U_{y}=3.5\;m/s$ at the time instant of t=1.17 s.

The effect of the staggered distance on the trailing side, represented by $\epsilon_{R}$ (Figure 16), on the tobacco-stem particle segregation is analyzed. As shown in Figure 16, increasing $\epsilon_{R}$ promotes greater transport of tobacco particles toward the stem collector.

**Figure 17:**
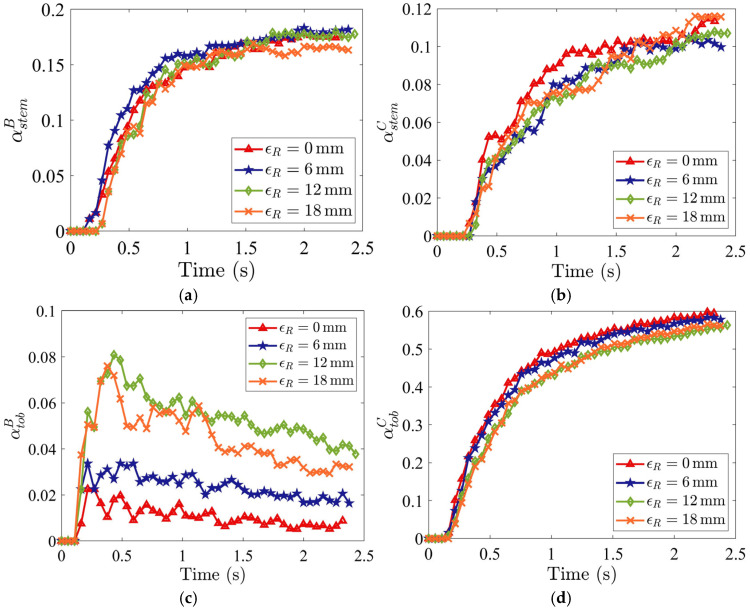
Time evolutions of mass fractions (**a**) $\alpha_{stem}^{B}$ (stem in stem collector), (**b**) $\alpha_{stem}^{C}$ (stem in tobacco collector), (**c**) $\alpha_{tob}^{B}$ (tobacco in stem collector), and (**d**) $\alpha_{tob}^{C}$ (tobacco in tobacco collector), for different staggered distances on the trailing side $\epsilon_{R}$ where $U_{x}=5.0\;m/s\;and\;U_{y}=3.5\;m/s$.

The stem particle mass fraction in Region B, $\alpha_{stem}^{B}$, is smaller at the largest $\epsilon_{R}$ (18 mm) and remains nearly constant for other values of $\epsilon_{R}$ (Figure 17a), while the stem particle mass fraction in Region C, $\alpha_{stem}^{C},$ is almost independent of $\epsilon_{R}$ (Figure 17b). The tobacco particle mass fraction in Region B, $\alpha_{tob}^{B}$, generally increases with increasing $\epsilon_{R}$ (Figure 17c), causing a greater loss of tobacco particles. A higher tobacco particle mass fraction in Region C, $\alpha_{tob}^{C}$, is obtained with a smaller $\epsilon_{R}$ (Figure 17d). As a result, the zero staggered distance on the trailing side (i.e., $\epsilon_{R}=0$) provides the best segregation performance with minimal tobacco loss to Region B, and most of the tobacco particles are collected in Region C. At a narrow channel width ($\epsilon_{R}=18$), particles frequently collide with the protruding channel edge, lose horizontal momentum, and are diverted into the stem collector, where the current upward airflow is insufficient to re-entrain them. In contrast, for smaller $\epsilon_{R}$ (wider channel width), no protruding wall impact occurs, so tobacco particles retain momentum and are carried by the horizontal flow ($U_{x})$ to the tobacco collector.

#### 4.7.2. Staggered Distance on Leading Side $\epsilon_{L}$

**Figure 18:**
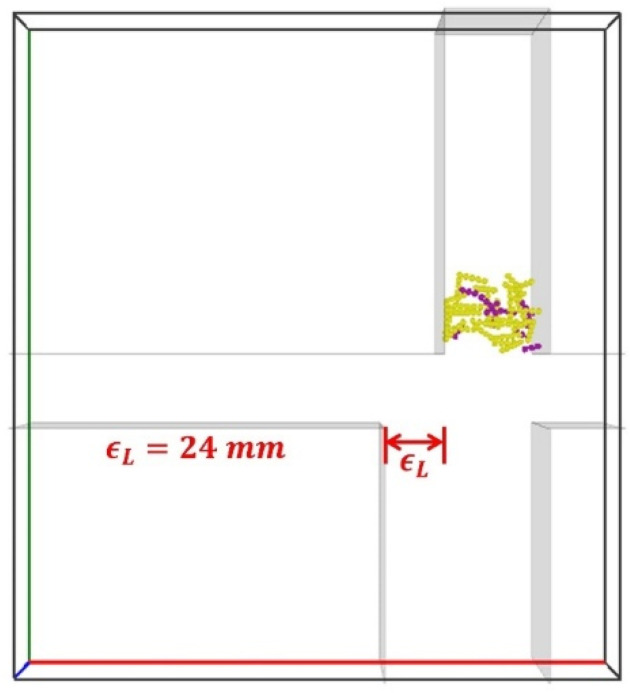
Representation of staggered distance on the leading side $\epsilon_{L}$.

The impact of staggered distances on the leading side $\epsilon_{L}$ (illustrated in Figure 18) on the segregation is investigated here. An increase in $\epsilon_{L}$ results in a wider stem collector, allowing more vertical air to flow into the domain from the bottom and increasing the chances for the particles falling into the stem collector.

**Figure 19:**
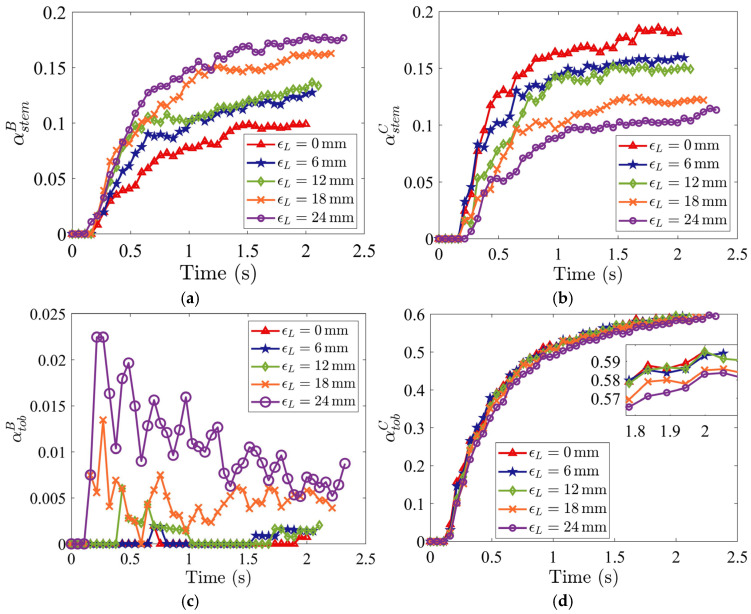
Time evolutions of mass fractions (**a**) $\alpha_{stem}^{B}$ (stem in stem collector), (**b**) $\alpha_{stem}^{C}$ (stem in tobacco collector), (**c**) $\alpha_{tob}^{B}$ (tobacco in stem collector), and (**d**) $\alpha_{tob}^{C}$ (tobacco in tobacco collector), for different staggered distances on the leading side $\epsilon_{L}$. The inlet air velocities are specified as $U_{x}=5.0\;m/s\;and\;U_{y}=3.5\;m/s$, and the staggered distance on the trailing side is $\epsilon_{R}=0\;mm$.

As $\epsilon_{L}$ increases, stem particle mass fraction in Region B, $\alpha_{stem}^{B}$, increases steadily (Figure 19a); meanwhile, the tobacco particle mass fraction in Region B, $\alpha_{tob}^{B}$, is negligible for $\epsilon_{L}\leq 12\;mm$ (Figure 19c), and $\alpha_{tob}^{B}$ increases with $\epsilon_{L}$ for $\epsilon_{L}>$ 12, leading to a bigger loss of tobacco particles. However, the stem particle mass fraction in Region C, $\alpha_{stem}^{C}$, decreases with the increase in $\epsilon_{L}$ (Figure 19b), improving the purity in the tobacco collector. The tobacco particle mass fraction in Region C, $\alpha_{tob}^{C}$, is insensitive to the variations in $\epsilon_{L}$ (Figure 19d). Thus, an intermediate distance of around $\epsilon_{L}=12$ mm provides optimal segregation with minimal tobacco loss and a lower concentration of stem particles in the tobacco collector.

#### 4.7.3. Height of Horizontal Channel $H_{w}$

**Figure 20:**
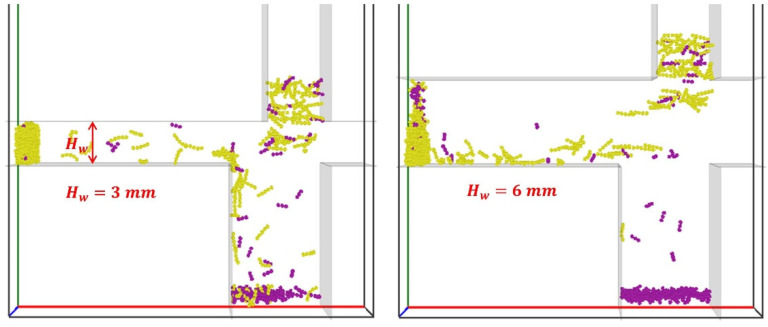
Snapshots of tobacco-stem particle segregation for two different heights of horizontal channel,$\;H_{w},$ with $U_{x}=5.0\;m/s\;and\;U_{y}=3.5\;m/s$, staggered distances of $\epsilon_{R}=0\;mm$ and $\epsilon_{L}=24\;mm$, at the time instant of t=1.86 s.

Two different heights of the horizontal channel ($H_{w}$ = 3 mm and 6 mm) are considered in the simulations. It can be seen in Figure 20 that with a larger height of $H_{w}\;=$ 6 mm, fewer tobacco particles (yellow) fall into Region B (stem collector), while more stem particles (magenta) transfer into the horizontal channel and reach the tobacco collector.

**Figure 21:**
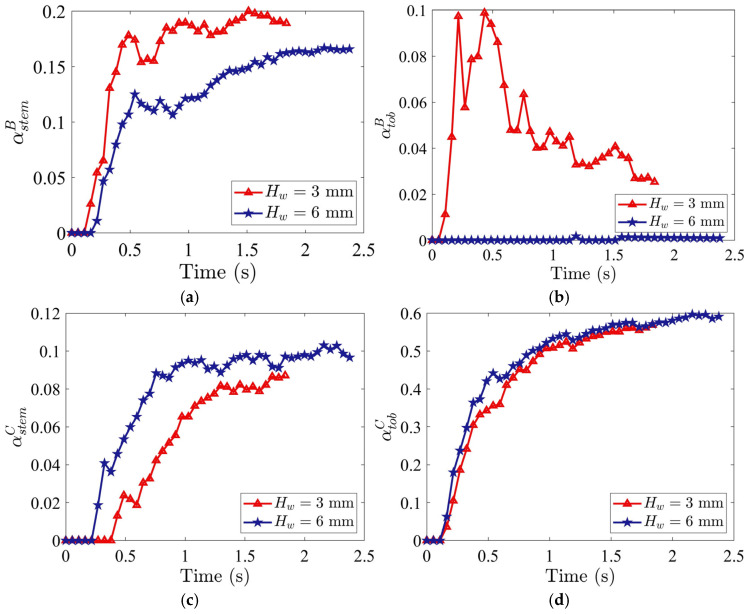
Time evolutions of mass fractions (**a**) $\alpha_{stem}^{B}$ (stem in stem collector), (**b**) $\alpha_{tob}^{B}$ (tobacco in stem collector), (**c**) $\alpha_{stem}^{C}$ (stem in tobacco collector), and (**d**) $\alpha_{tob}^{C}$ (tobacco in tobacco collector), for two different heights of horizontal channel, $H_{w},$ with air inlet velocities of $U_{x}=5.0\;m/s\;and\;U_{y}=3.5\;m/s$, and staggered distances of $\epsilon_{R}=0\;mm$ and $\epsilon_{L}=24\;mm$.

The stem particle mass fraction in Region B, $\alpha_{stem}^{B}$, increases over time for both $H_{w}$, and the rate of increase is steeper for $H_{w}=6\;mm$ (Figure 21a), while collection of tobacco particles in Region B, $\alpha_{tob}^{B}$, is negligible (Figure 21b), indicating that a horizontal channel height of 6 mm is more efficient in reducing tobacco particle loss. In contrast, the stem particle mass fraction in Region C, $\alpha_{stem}^{C}$, is higher over time at $H_{w}=6\;mm$ but becomes less pronounced after t=1.5 s (Figure 21c). The tobacco particle mass fraction in Region C, $\alpha_{tob}^{C}$, is also slightly higher with $H_{w}=6\;mm$ (Figure 21d). Thus, increasing the height of the horizontal channel is analogous to increasing the horizontal inflow air velocity $U_{x}$, and reducing tobacco particle loss in Region B, but it also increases stem particle mass fraction in Region C, degrading the purity.

### 4.8. Summary of Optimal Separation Conditions

For a quantitative overview of the optimal separation performance, Table 3 is provided to summarize the conditions yielding the highest F1 score for each key parameter studied, along with the corresponding purity and recovery values. The F1 score serves as a single objective metric to identify the best compromise between high tobacco purity and high tobacco recovery in Region C. As shown in Table 3, the highest overall F1 score (87.2%) is achieved with a stem volume fraction of $C_{s}$ = 8.5%, corresponding to purity and recovery of 91.2% and 83.6%, respectively. Optimal performance is obtained at the air inflow velocities of $U_{x}\;=\;6.0\;m/s$ and $U_{y}\;=\;3.5\;m/s$. Lower initial packing density (ϕ = 4%) and staggered distances of $\epsilon_{R}\;=\;0\;mm$ and $\epsilon_{L}\;=\;24\;mm$ optimize the separation.

## 5. Conclusions

This study analyzes the segregation of flexible rod-like particle mixtures by intersecting airflow in an air separator using coupled DEM-CFD, with tobacco and stem particles used as model materials. The key findings are summarized as follows:

- Segregation depends significantly on both inflow air velocities that govern the trade-off between tobacco loss and stem contamination. Smaller $U_{x}$ or $U_{y}$ increases tobacco loss to the stem collector, while larger $U_{x}$ or $U_{y}$ improves recovery but reduces purity. Optimal values are $U_{x}=6.0\;m/s$ and $U_{y}=3.5\;m/s$.
- A higher initial solid volume fraction (Φ) results in poorer segregation, with more stems translating into the tobacco collector, and more tobacco particles falling into the stem collector. This deterioration is caused by the increased particle collisions that restrict relative particle motion and thus separation. A higher stem particle volume fraction, $C_{s},$ also increases tobacco loss. The best performance is obtained at the lowest tested ϕ=4% and $C_{s}=8.5\%$.
- Flow field geometry affects air–particle interactions and separation performance. Increasing the trailing side staggered distance $\epsilon_{R}$ reduces tobacco recovery, while increasing the leading side staggered distance $\epsilon_{L}$ improves tobacco purity but reduces recovery. Optimal performance is achieved at $\epsilon_{R}=0\;mm$ and $\epsilon_{L}=24\;mm$.
- Increasing horizontal channel width $H_{w}$ enhances airflow flux in the segregation region and significantly reduces tobacco loss to the stem collector, with only a slight increase in stem contamination. However, $H_{w}=\;6\;mm$ yields a higher F1 score, indicating this configuration is favorable for enhancing overall separation efficiency.

Thus, based on the quantitative evaluation of tobacco purity and recovery, an optimal setting of the parameters (mentioned in the above bullet points) is identified for the present separator configuration, which achieves the best balance between separation purity and product recovery. It is expected that, as an air separator has a flow geometry similar to that in this study, the separation performance varying with these key parameters (the inflow air velocities, solid volume fraction, and geometric dimensions) follows the same trend. However, the observed separation findings are valid within the ranges of the parameters considered in this work (as described in Table 1 and Table 2), and extrapolated applications beyond the present ranges are not guaranteed.

Some limitations in the present numerical studies exist: (i) only a small scale of the air separator is established due to the extremely high computational cost, and a more comprehensive scale-up law is needed to extend the present results for the prediction of real, large-scale processes; (ii) present rod-like particles are simplified models of irregular-shaped biomass particles, and simulations with realistic irregular-shaped particles may improve the accuracy of the numerical results; and (iii) wet biomass particles experience cohesive, liquid-bridge forces in a moist environment, and cohesive interparticle contacts should be considered to explore the separation of such wet particles.

## Figures and Tables

**Table 1:** Properties and boundary conditions of the airflow.

Air Parameters	Values
Bed dimensions, x×y×z $\left(mm^{3}\right)$	246×270×24
$\mathrm{Initial}\;\mathrm{air}\;\mathrm{density}\;(kg/m^{3})$	1.204
Initial air pressure (Pa)	101,325.0
Temperature (K)	293.0
Bulk viscosity (Pa.s)	0.0
Shear viscosity (Pa.s)	$1.8\times 10^{-5}$
$\mathrm{Horizontal}\;\mathrm{inflow}\;\mathrm{air}\;\mathrm{velocity},\;U_{x}$ (m/s)	2.0–6.0
$\mathrm{Vertical}\;\mathrm{inflow}\;\mathrm{air}\;\mathrm{velocity},\;U_{y}$ (m/s)	1.0–3.7
$\mathrm{Fluid}\;\mathrm{cell}\;\mathrm{dimensions}\;(mm^{3})$	6×6×6
Fluid cell size (mm)	6
Number of fluid cells	7380

**Table 2:** Properties of the particles used in the present study.

Properties	Tobacco Particle	Stem Particle
Length (mm)	5.4 (*AR* = 2), 13.5 (*AR* = 5), and 21.6 (*AR* = 8),	8.1
Diameter (mm)	2.7	2.7
Particle aspect ratio, AR	2, 5, and 8	3
$\mathrm{Material}\;\mathrm{density}\;(kg/m^{3})$	264	639.8
Poisson’s ratio, ζ (−)	0.2	0.2
Elastic modulus for fiber-fiber contact, Ec (Pa)	1.0 × 10^10^	1.0 × 10^10^
$\mathrm{Elastic}\;\mathrm{modulus}\;\mathrm{for}\;\mathrm{bond}\;\mathrm{tension}/\mathrm{compression},\;E_{b}^{a}$ (Pa)	1.0 × 10^10^	1.0 × 10^10^
Elastic modulus for bond bending, Eb (Pa)	4.4 × 10^3^	5.0 × 10^8^
Shear modulus for bond shearing and twisting, Gb (Pa)	1.83 × 10^3^	2.08 × 10^8^
Contact damping coefficient, βc (−)	1.63 × 10^−2^	1.63 × 10^−2^
Bond damping coefficient, βb (−)	3.35 × 10^−2^	3.35 × 10^−2^
$\mathrm{The}\;\mathrm{friction}\;\mathrm{coefficient}\;\mathrm{between}\;\mathrm{particles},\;\mu_{pp}$ (−)	0.45	0.45
$\mathrm{The}\;\mathrm{friction}\;\mathrm{coefficient}\;\mathrm{between}\;\mathrm{particles}\;\mathrm{and}\;\mathrm{wall},\;\mu_{pw}$ (−)	0.59	0.59
Volume fraction of stem particles in the mixture (−)	8.5% , 12.75% , 17% (base), 21.75% , and 25.5%	
Initial packing density of particle mixture (−)	4% , 8% (base), and 16%	

**Table 3:** Optimal separation parameters and corresponding performance metrics.

Parameter	Optimal Value	Purity (%)	Recovery (%)	F1 Score (%)
$U_{x}$	6.0 m/s	83.2	84.2	83.7
$U_{y}$	3.5 m/s	84.5	80.9	82.7
ϕ	4%	92.1	79.2	85.2
$C_{s}$	8.5%	91.2	83.6	87.2
$\epsilon_{R}$	0 mm	84.5	82.5	83.5
$\epsilon_{L}$	24 mm	84.5	82.5	83.5

## Data Availability

The original contributions presented in this study are included in the article. Further inquiries can be directed to the corresponding author.
